# Development of an Insecticide-Free Trapping Bednet to Control Mosquitoes and Manage Resistance in Malaria Vector Control: A New Way of Thinking

**DOI:** 10.3390/insects11110732

**Published:** 2020-10-26

**Authors:** Chouaibou S. Mouhamadou, Kun Luan, Behi K. Fodjo, Andre J. West, Marian G. McCord, Charles S. Apperson, R. Michael Roe

**Affiliations:** 1 Centre Suisse de Recherches Scientifiques en Côte d’Ivoire (CSRS), 01BP, Abidjan 1301, Cote d’Ivoire; behikouadio@gmail.com; 2 Department of Entomology and Plant Pathology, Campus Box 7647, 3230 Ligon Street, North Carolina State University, Raleigh, NC 27695, USA; apperson@ncsu.edu (C.S.A.); mroe@ncsu.edu (R.M.R.); 3 College of Natural Resources, Campus Box 8001, 2820 Faucette Drive, North Carolina State University, Raleigh, NC 27695, USA; kluan@ncsu.edu (K.L.); marianmccord@gmail.com (M.G.M.); 4 Department of Textile and Apparel, Technology and Management, North Carolina State University, Raleigh, NC 27695, USA; ajwest2@ncsu.edu; 5 Comparative Medicine Institute, North Carolina State University, Raleigh, NC 27695, USA

**Keywords:** malaria, vector control, insecticide resistance, long-lasting bednet, vector control failure, trapping bednet, permanet 2.0

## Abstract

**Simple Summary:**

Insecticide resistance in mosquitoes reduces the effectiveness of malaria control interventions and has reversed the gains made in reducing malaria morbidity. Hence, new strategies are needed to mitigate the spread of resistance, preserve the efficacy of available insecticides, and restore the effectiveness of control. To combat resistance to insecticides in malaria mosquitoes, WHO recommends using long-lasting insecticide-impregnated mosquito bednets (LLINs) as well as the synergist piperonyl-butoxide (PBO). PBO enhances the insecticidal effect of the treated bednet. Unfortunately, decreases in performance of PBO-LLINs are now reported in some regions of Africa where mosquitoes are resistant to insecticides. Our objective was to develop an insecticide-free, mechanical solution that kills mosquitoes regardless of their insecticide resistance status, ultimately overcoming the problem of insecticide resistance. We designed and developed an insecticide-free mosquito trapping bednet for mass mosquito trapping and killing, the “T-Net”, and we show its efficacy compared to a conventional LLIN in Africa. Mathematical models were also developed to predict T-Net efficacy in individual homes and at the community level.

**Abstract:**

Mosquito-borne malaria kills 429,000 people each year with the problem being acute in sub-Saharan Africa. The successes gained with long-lasting pyrethroid-treated bednets are now in jeopardy because of wide-spread, pyrethroid resistance in mosquitoes. Using crowd modeling theory normalized for standard bednet architecture, we were able to design an attract–trap–kill technology for mosquitoes that does not require insecticides. Using three-dimensional polyester knitting and heat fixation, trap funnels were developed with high capture efficacy with no egression under worst-case laboratory conditions. Field testing in Africa in WHO huts with Gen1-3 T (trap)-Nets validated our model, and as predicted, Gen3 had the highest efficacy with a 4.3-fold greater trap–kill rate with no deterrence or repellency compared to Permanet 2.0, the most common bednet in Africa. A T-Net population model was developed based on field data to predict community-level mosquito control compared to a pyrethroid bednet. This model showed the Gen3 non-insecticidal T-Net under field conditions in Africa against pyrethroid resistant mosquitoes was 12.7-fold more efficacious than single chemical, pyrethroid-treated nets.

## 1. Introduction

Malaria is the leading cause of morbidity and mortality in sub-Saharan Africa with 212 million cases annually estimated by the WHO and about 429,000 deaths each year [1]. Malaria prevention is mainly based on vector (mosquito) control, using insecticide indoor residual spraying (IRS) or long-lasting insecticide-impregnated mosquito bednets (LLINs) [2]. The efficacy of these measures depends primarily on the susceptibility of the mosquito to insecticides. An estimated 663 million cases of malaria have been averted in sub-Saharan Africa since 2001 as a result of the scale-up of malaria control interventions; 69% of these reduced cases were a direct result of the use of LLINs [3]. Unfortunately, mosquitoes have, in recent years, become resistant to insecticides including the pyrethroids, the only chemistry approved for bednets [4,5,6]. This resistance is threatening the efficacy of chemical-based, vector control [7,8,9]. There is an urgent need to develop new strategies to mitigate this problem.

Development of a new insecticide for bednets that is safe to human exposure every night for years, that must survive human handling each day and periodic washing, and that will not promote mosquito resistance and cross-resistance, is a challenge [10]. One solution is the reformulation of agricultural chemicals with completely different modes of action to that of the pyrethroids. This is a challenge since adult mosquitoes have been exposed to these insecticides applied to crop plants and as larvae developing in agricultural runoff; it has been hypothesized that mosquito resistance to bednets is a result of pesticide exposure from agricultural systems more than actual bednet use [11].

These challenges have led us to a different approach for mosquito control with bednets, a mechanical trapping and killing device. It is well known that mosquitoes are attracted to carbon dioxide gas and other human odors that are emitted from a warm body sleeping under the net, and these attractants rise through the top of the bednet [12]. Infrared video tracking has shown that about 75% of mosquitoes follow this odorant plume and are attracted to the top surface of the bednet [13,14]. We have taken advantage of this natural behavior to develop a mosquito trapping bednet, the T-Net, designed to trap and kill susceptible and resistant mosquitoes. The non-insecticidal T-Net has (i) a lower sleeping compartment and (ii) an upper mosquito trap compartment with funnels on the trap roof as mosquito entry points into the trap.

To optimize trapping performance, we hypothesized that mosquito movement is random outside of the trap compartment in the absence of attractants and visual cues. If we then consider mosquitoes as ideal gas particles, we can use the Maxwell–Boltzmann distribution [15] to develop a mathematical model to optimize the number, size, and positioning of funnels to maximize trapping efficacy that would also apply when someone is sleeping under the net. Different T-Nets derived from the model were assayed in walk-in simulated WHO huts using human subjects [16] and once proof of principle was demonstrated, further validated with wild-type, insecticide-resistant *Anopheles gambiae* mosquitoes under field conditions in Tiassale, Côte d’Ivoire, Africa. From the field data, we developed a second model to predict community-level mosquito control by the T-Net versus the most common bednet deployed in Africa.

## 2. Materials and Methods

### 2.1. T-Net Construction

The non-insecticidal T-Net has (i) a lower sleeping compartment and (ii) an upper mosquito trap compartment with funnels on the trap roof (with the small opening of the cone(s) pointing down into the trap compartment). The funnels serve as mosquito entry points into the trap. The sleeping compartment of the T-Net was a standard (single-sleeper), non-insecticidal bednet (Huzhou Shuanglu Knitting Mill Co., Ltd., Jinhua, Zhejiang, China). The trap compartment was constructed from a second non-insecticidal bednet that was sewn on the original bednet roof (open end down) but where the height of the sleeping compartment (which is now the trap compartment) is reduced. Then knitted cones described later were sewn into holes made in the roof of the trap compartment. The detailed architecture of the T-Net and different iterations used in our research are discussed later. Once mosquitoes enter into the trap compartment through the funnels, they are blocked from reaching the sleeper by the roof of the first bednet.

### 2.2. Cone Knitting

T-Net prototyping was conducted using different knitted cones sewn into the roof of the trap compartment. Knitted cones can easily be constructed in any shape and size, are easily sewn into our T-Net, and can be folded into a flat, compact package for storing and shipping of bednets. Cones were knitted on a flat, computerized knitting machine, SWGN2 (Shima Seiki, Sakata Wakayama, Japan), where their dimensions for prototyping are easily changed by standard program options. Details on cone sizes are discussed later. The gauge of knitting machine was selected so that the resulting cone fabric would have openings between loops similar to those of traditional bednets. Like the construction of stockings or socks, cones were knitted in-the-round to make a 3D shape. Use of this construction method allowed us to alter the shape, depth, width, and height in all sections of the cone to meet our design demands. The knitted cones had three main parts—a large opening at the mouth (which was designed to allow for passage of mosquitoes into the cone), tapered cone walls (which were designed to direct the movement of mosquitoes into the trapping compartment), and a neck opening into the trapping compartment (the size of the neck was optimized to allow passage of mosquitoes and minimize egression from the trap). To prevent collapsing and flattening of the knitted cones, they were constructed with thermal bond fibers. This yarn is a polyester binder-spun yarn in which each fiber is composed of a sheath part (co-polyester) and a core (regular polyester). The bursting strength and durability of these cones described later were compared to conventional bednet material to make sure they would stand up to years of use.

### 2.3. Cone Bursting Strength and Durability Measurements

A burst test (James H. Heal TruBurst 2, ASTM D3786, James H. Heal & Co. Ltd., Halifax, UK) was used to measure the durability of the cones constructed versus bednet fabric; this is achieved by increasing the hydrostatic pressure across the textiles until they ruptured (Figure S1). The pressure was applied to a circular region of the fabric via an elastic diaphragm. The fabric sample was firmly held around the circular edge by pneumatic clamping. When the pressure was applied, the fabric deformed together with the diaphragm. The bursting strength corresponded to the maximum pressure supported by the fabric before failure.

The abrasion test (Figure S2) was used to assess the cone textile durability by friction compared to the bednet fabric. Abrasion resistance refers to the resistance of a fabric in the process of repeated friction with a defined, rough surface. To run the test, the fabric was loaded onto the lower plates of the abrasion tester (Maxi Martindale 1609, ASTM D4966, James H. Heal & Co. Ltd., Halifax, UK), and then abraded using oscillating circles. The assay end point was the duration and number of circles until the first hole appeared.

### 2.4. Tunnel Test for Assessment of Cone Efficiency

A modified WHO-tunnel test [17] was conducted to assess the rate at which mosquitoes would move through knitted cones and their potential use as part of a trap mechanism. The tunnel test was modified to include three compartments—a top compartment which operated as a mosquito release chamber, a middle compartment which operated as a trapping container, and a lower compartment that contained the arm of a human subject to attract host-seeking mosquitoes. The top and middle compartments were separated by the knitted cone, and the middle and lower compartments were separated by a polyester net fabric to prevent the human subject from receiving mosquito bites (NCSU IRB approved protocol 16897).

The tunnel test was placed vertically to mimic the position of a cone on the top of the trap compartment, with the small opening of the cone pointing down. A human arm cleaned with distilled water and then air dried was inserted into the bottom compartment. Two replicates (the first one of 106 mosquitoes and the s using 50 depending on availability) of unfed five to eight-day-old, host seeking *An. gambiae* Kisumu-strain female adult mosquitoes were released into the top compartment. Mosquitoes used were reared in the Dearstyne Laboratory at North Carolina State University (Raleigh, NC) according to MR4 rearing protocols [18]. After 2 h from the time of release, the mosquitoes that remained in the top chamber were removed using a mouth aspirator and counted. The human arm was removed from the bottom compartment, and trapped mosquitoes were observed for an additional 2 h in the middle compartment. Mosquitoes moving from the middle to the top compartment was scored as egression.

### 2.5. Laboratory Walk-In Hut Trials

Laboratory walk-in hut trials were conducted in the Dearstyne Entomology Building. The rooms were 1.58 × 2.18 × 2.03 m (depth × width × height) and the tests conducted at 27 ± 1 °C and 65 ± 4% relative humidity. The T-Net was set up above a portable bed with the bottom of the sleeping compartment of the net on the floor of the room. Trapping was assessed with a human subject in the sleeping position under the net (NCSU IRB approved protocol 9067). Five- to eight-day old, unfed *An. gambiae* Kisumu-strain, adult female mosquitoes (described earlier) were used for these studies. For each test, the mosquitoes were released into the room but outside of the bednet. The number of mosquitoes released varied between 40 and 100 to determine if population density in the room had any effects on trap efficacy. The total number of replicates was six and these were conducted on different days. Trapping was conducted for 3 h during the photophase, since this mosquito strain has, for years, been fed with lights on. At the end of the experiment, mosquitoes were vacuum-collected from outside of the trap compartment (in the room) and counted immediately. To assess egression, trapped mosquitoes were not removed from the trap compartment. The number remaining in the trap were then counted after 30 min, 1 h, 2 h, 6 h, and overnight.

### 2.6. Field Trials in Africa

The study was conducted in experimental huts according to WHO protocols [17] in the municipality of Tiassalé (5°53′54” N et 4°49′42” W) in November 2018. The site is located in the south of Côte d’Ivoire, about 110 km north of the country’s major city, Abidjan. The climate is tropical and characterized by four seasons—a long rainy season (March–July) during which two thirds of the annual rainfall occurs, a short dry season (July–August), a short rainy season (September–November), and a long dry season (December–March). The average annual rainfall is 1739 mm with an average annual temperature of 26.6 °C. The annual average relative humidity is around 70%. Rice production occurs in the lowlands of Tiassalé which facilitates the proliferation of mosquitoes throughout the year, and malaria is the leading cause of morbidity in the local population. The transmission of the disease is mainly due to *Anopheles coluzzii* (80%) and *An. gambiae* (20%) which have developed multiple resistances to insecticides [19,20].

The experimental field station of Tiassalé is made up of 18 standardized experimental huts (described in more detail later) situated close to the rice plantations. Each hut is 2.5 m long, 1.75 m wide, and 2 m high. The walls are made of concrete bricks and plastered with cement, while the floor is made of cement and the roof of corrugated iron sheets. A plastic cover is mounted underneath the roof as a ceiling to facilitate manual collection of mosquitoes. Each hut is built on a platform made of concrete surrounded by a water-filled moat that prevents entry of foraging ants. Entry of mosquitoes is facilitated through four window slits located on three sides of the hut. The slits are designed in such a way as to prevent mosquitoes from escaping once they are inside the hut. Each hut is equipped with a veranda trap located on the fourth side, made of sheeting and screening to capture mosquitoes that would otherwise escape.

Four sleepers were trained and paid to sleep under the bednets. Each day, both sleepers and bednets were randomly positioned between four different huts. Trapping was conducted from 21:00 to 5:00, and the mosquitoes collected each morning after the sleep cycle was completed. Sleepers and bednets were rotated on a daily basis according to two Latin-square tables (Figure S3). The trial was conducted for 14 d. Each day, after mosquito collections, the huts were cleaned and the bednets rotated appropriately. Prior to the trial, sleepers were all vaccinated against yellow fever and were taken to the hospital to check for malaria parasites. All sleepers gave informed consent prior to enrolment in the study (CSRS IRB protocol #02-2011/MSLS/CNER-P). Within the hut, resting and dead non-trapped mosquitoes were collected from the room and in the veranda trap using 5 mL (12 mm × 75 mm) glass hemolysis tubes. Trapped mosquitoes were removed with mouth aspirators from the T-Net trap compartment by the field technicians. All mosquitoes were taken to the field laboratory and identified to genus level and scored as trapped-dead, trapped-alive, not trapped-dead, or not trapped-alive. Live mosquitoes were placed in cups and given access to a sugar solution for 24 h in the insectary at 25–27 °C and 70–80% relative humidity to assess delayed mortality.

Three different insecticide-free T-Nets (described in detail later) were evaluated in comparison to the WHO-recommended and widely-used Permanet 2.0-LLIN (PN2.0), a positive control. The PN2.0 was brand new and provided by the National Malaria Control program in Cote d’Ivoire.

The Kruskal–Wallis non-parametric test with the Conover–Iman multiple pairwise comparisons and the Bonferroni correction were used to analyze the data (alpha = 0.05). The analyses were conducted using the XLSTAT software package version 2019.4.1. [21] The overall blood-feeding rate was less than 3%.

## 3. Results

### 3.1. Crowd Model Applied to Mosquito Trapping

**Figure 1:**
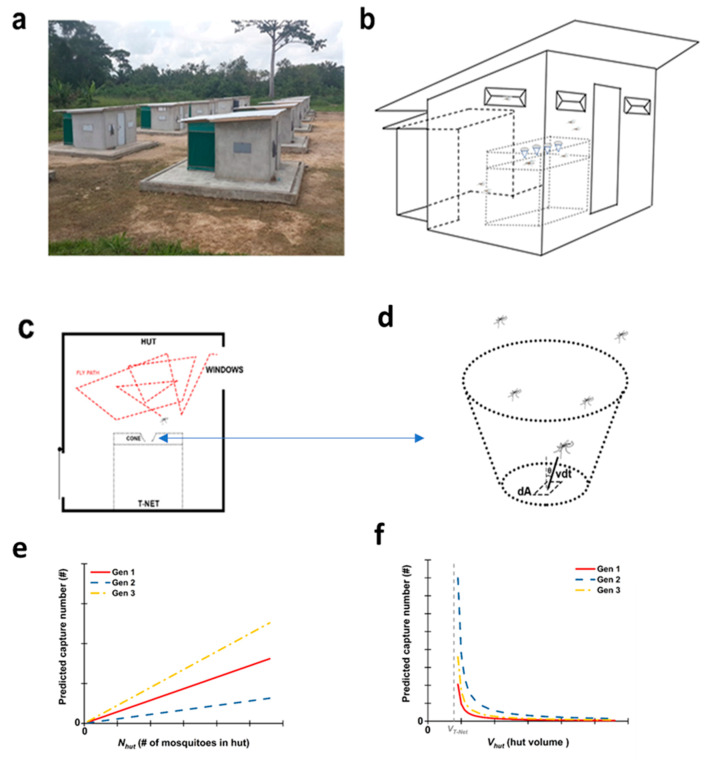
Principle and model of the T-Net. (**a**) Picture of experimental huts in the region of Tiassale in Cote d’Ivoire (Africa), where the field trials were conducted; (**b**) schematic of the hut and the T-Net deployed in the hut; (**c**) schematic of the mosquito flight track in the hut; (**d**) unit area on the bottom of the T-Net cone that captures the mosquito; (**e**) predicted capture number for Gen1, Gen2, and Gen3 T-Nets (see Figure 2a–c, respectively) with an uncertain number of mosquitoes flying into the hut; and (**f**) the predicted capture number for Gen1, Gen2, and Gen3 T-Nets when hut volume increases and the number of mosquitoes flying into the hut is constant.

In our effort to develop a non-insecticidal bednet that kills mosquitoes by trapping, preliminary proof of concept studies was conducted to model mosquito interactions with a bednet designed for trapping. The model, if predictive of field conditions, could be used to evaluate different trap designs and make a better mosquito trap. The model could also provide a better understanding of how mosquitoes interact with bednets. If we treat a mosquito as an ideal gas particle, and the flight track is assumed to be random, the Maxwell–Boltzmann distribution [15] can be used for defining the random flight of mosquitoes at different velocities. This can further be applied to the dimensions of a standardized WHO approved, experimental research hut [16] as shown in Tiassale, Cote d’Ivoire (Africa) where our field work was conducted (Figure 1a,b). Figure 1c shows the process by which mosquitoes can fly from the interior of the hut through knitted cones into a trap compartment mounted above the sleeping compartment (Figure 1b). Mosquitoes that are in the trap compartment are blocked from the sleeper by the trap bottom, which is also the sleep compartment roof. Once the mosquitoes are trapped, they become exhausted and quiescent, and die from dehydration a few hours later.

**Figure 2:**
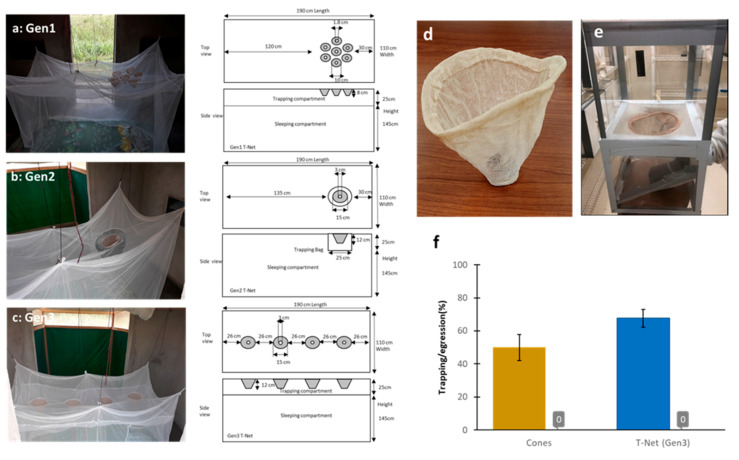
Different T-Net designs and testing. (**a**) Generation (Gen) 1 T-Net made of a circular aggregate of seven small cones, (**b**) Gen2 T-Net made with one large cone fitted on a trapping bag (**c**) Gen3 T-Net fitted with four large cones, (**d**) example of a knitted cone, (**e**) experimental tunnel apparatus to assess the trapping efficacy of a knitted cone, and (**f**) results obtained from the tunnel test for trapping efficiency of a large cone (on the left) and for the Gen3 T-Net in a laboratory hut experiment (on the right). The histograms represent the trapping rates, the bars on top are the 95% confidence intervals, and “0” are the exit rates (= egression) in the absence of host cues.

Knitted cones were developed as access points to the trap compartment (Figure 1b and Figure 2) with superior durability to standard bednet fabric (Figures S1 and S2). Use of knitting to make these cones allowed us to study different shapes, depths, widths, and heights to meet our design demands. The knitted cones had three main parts—a large opening at the mouth (which was designed to allow for passage of mosquitoes into the cone), tapered cone walls (which were designed to direct the movement of mosquitoes into the trapping compartment), and a neck opening into the trapping compartment (the size of the neck was optimized to allow passage of mosquitoes and minimize egression from the trap).

For model development, we assumed that (i) the flight track of mosquitoes is random (Figure 1c), (ii) the diameters of the cone into the trap compartment are large enough not to affect the flight path of other mosquitoes, and (iii) the trapping does not perturb the flight velocity of the remaining mosquitoes in the hut. Figure 1d assumes a hole area dA, a mosquito at a distance vdt from the hole, moving at a speed v and at an angle θ from the normal surface toward the area dA. All mosquitoes within a parallelepiped volume around the dA area moving toward the hole with speed v will pass through the top opening of the cone in the time interval dt. Therefore, the total number of mosquitoes flying through the dA area in the time interval dt is as follows:

(1) $$N_{A}=\rho vcos\theta dAdt$$

where ρ is the mosquito density in the hut.

Assuming the distribution of individual mosquito velocities obeys the Maxwell–Boltzmann distribution, which was first defined and used for describing particle speeds in idealized gases, one integrated expression of the distribution relates particle density with average velocity. Similarly, the average trapped mosquito number per area per time is then:

(2) $$\dot{N}=C\bar{v}\rho$$

where $\bar{v}$ is the average flight speed, ρ is the mosquito density in the hut, and C is a constant determined by the active behavior of the mosquito (1/4 in ideal gas theory). As mentioned above, most of mosquitoes fly to the top of the bednet and thus the work volume is as follows:

(3) $$V_{w}=V-V_{net}-V_{s}$$

where V is the hut volume, $V_{net}\;$is the bednet volume, and $V_{s}$ is the volume surrounding the bednet (except the volume on top of the bednet).

Assuming mosquitoes are distributed evenly on the top of the bednet, the trapped mosquito number for the T-Net in the testing time t is as follows:

(4) $$N=C\times a\times \frac{N_{hut}\times \bar{v}}{\left(V-V_{net}-V_{s}\right)}\times t$$

where $N_{hut}$ is the total mosquito number in the hut, C is a constant, and a is the bottom area of the cone. Thus, the T-Net model relates trapping number with container volume and flight velocity. If the number $N_{hut}\;$of mosquitoes in a hut is a variable and other parameters are constant, the predicted results of Equation (4) are shown in Figure 1e and if the volume $V_{hut}$ of the hut is a variable and the number of mosquitoes a constant, the predicted trapping number for the T-Net is shown in Figure 1f for three different versions of the net that we investigated for model development and field validations (i.e., Gen1–3 prototypes shown in Figure 2a–c, respectively).

### 3.2. Construction of Different T-Nets and Model Prediction

The attraction of mosquitoes through the funnels into the trap compartment is hypothesized to be from insect attraction to carbon dioxide and other odors from human respiration. If this is the case, funnels should only be needed on the head end of the net. Based on this assumption, we designed and constructed the Generation (Gen) 1 T-Net with a circular aggregate of 7 cones (each cone having a 10 cm opening, an 8 cm depth, an inclination angle of 32 degrees, and a 1.8 cm small diameter); these cones were sewn around the midline of the long axis of the trap roof (a 25-cm-deep trap compartment), 30 cm from the sleeping end of the net (shown in Figure 2a). The model (4) predicted trapping rates shown by the regression curves in Figure 1e,f. To potentially increase the trapping rate and add additional functionality, a Gen2 T-Net was constructed (Figure 2b). In this case, one large cone 15 cm in the large diameter, 12 cm deep, and 3 cm in the small diameter, with a 51.3 degree of inclination angle, was fitted into a cylindrical trapping bag 25 cm deep and 25 cm in diameter fixed as before on the bednet roof 30 cm from the edge. The idea of using a trapping bag and a single cone was to reduce material costs for construction, make it possible to reduce the head space between the sleeper and the bednet roof (effectively increasing the odorant concentration on the bednet top) and to add practical functionality to the bednet design; the Gen2 net design could be used to retrofit any non-trap bednet including those already deployed in homes. From Equation (4), we predicted that this actually reduced the catch rate as compared to Gen1 (Figure 1e,f).

Intuitively, having the cones just above the head of the sleeper should produce the maximum trap rate if only carbon dioxide from the subject’s breathing is at work. At the same time, reducing the overall cross-sectional area of the small openings of the cones and/or the number of cones might reduce the catch rate but also reduce the egression rate once mosquitoes were captured. There is also the possibility that odorants from the subject’s body could be in play in trapping and that spreading cones in different areas on the net top might improve capture rates. To address the above in part, we increased the number of cones from one in Gen2 (Figure 2b) to four in Gen3 (Figure 2c). The Gen3 T-Net had four large cones (15 cm large diameter, 12 cm deep, 3 cm small diameter, and 51.3 degree of inclination angle; the same dimensions as Gen2), where the cones were aligned lengthwise along the center line of the bednet with the cones spaced 26 cm apart. All cones opened into a 25-cm-deep trapping box (Figure 2c). Interesting, our model predicted an increase in the trapping rate over that for Gen1 and Gen2 based strictly on our model assumptions (Figure 1e,f).

Our model (4) predicted capture rates but not the possibility of egression once mosquitoes were trapped. Therefore, laboratory bioassays were conducted with lab-reared Kisumu strain, *An. gamibae*, adult females to examine egression rates across the large cones in the Gen3 net where the model predicted the highest capture rate. A high capture rate would not be optimal if the large cone and especially four large cones in Gen3 favored a higher egression rate. Studies were conducted using a modified WHO tunnel test [17] as shown in Figure 2e. The capture rate (with mosquitoes released into the top chamber and the subject’s arm as shown in the bottom chamber) was 49.5% after 2 h. More importantly, the egression rate once the mosquitoes were captured was 0% 2 h after the arm was removed (Figure 2f). These results suggested that even though we added a greater overall area for mosquito entry into the trap in Gen3 which our model suggested would increase capture rate, the increased capture rate predicted did not also increase egression. The tunnel tests were also a worst-case scenario where the host cues were removed, and the trap volume was much smaller than that for the Gen3 trap compartment.

Before going to the field in Africa to validate our trapping net model, a laboratory test was conducted with the Gen3 T-Net in a walk- in incubator (same dimensions as field huts, see details in the Materials and Methods section) and with a human subject under the net in the sleep position. Susceptible, laboratory-reared *An. gambiae* Kisumu were released into the room after the subject entered the sleeping compartment of the T-Net. A total of 300 unfed adult, host-seeking female mosquitoes were tested over six replicates with an average of 50 mosquitoes per replicate (range = 41–85 females). The trapping rate in these studies ranged from 45.28% to 86.90% after 3 h with an average capture rate of 67.7% (± 20.5%, 95% CI), (Figure 2f). No mosquito egression from the trapping compartment was observed at 30 min, 1 h, 2 h, 6 h, or overnight) for any of the replicates (Figure 2f). These results demonstrated proof of concept that the predicted best-performing Gen3 T-Net, under laboratory conditions simulating WHO experimental hut conditions at our field site in Africa, was efficacious in mosquito capture and retention.

### 3.3. Field Performance of Gen1, Gen2, and Gen3 T-Nets against Insecticide Resistant, Wild, Free-Flying Mosquitoes

To fully validate our model (4) for predicting the trapping efficacy for the T-Net, field trials were conducted comparing Gen1, Gen2, and Gen3 bednets that were compared to a positive control, the Permanent 2.0 (PN2.0)-LLIN (the most used LLIN in Africa). The other objective of the field trials was to evaluate the efficacy of the T-Nets compared to a popular LLIN (Permanet 2.0) and determine if the technology is reasonably practical as a mosquito control strategy or whether more research is needed to further optimize the T-Net. These studies were conducted in WHO-approved experimental huts [16] in the locality of Tiassale where wild *An. gambiae* malaria vectors are resistant to insecticides [19,20], with human subjects sleeping under the nets each night. Three entomological parameters [22,23] were measured: (i) the mean entry rate of mosquitoes per hut per night, (ii) the mean exit rate per hut each night, and (iii) the mean mortality rate per hut per night. For the T-Nets, we scored the trapped mosquitoes as alive or dead each morning but in practice, trapped mosquitoes are ecologically dead, i.e., they are quiescent, no longer able to blood-feed and typically die before the next sleep cycle. Only the *An. gambiae* s.l. were considered in this study. *Culex* spp. were also collected but in a low number (less than 100 total), too low for a valid separate analysis from that of *Anopheles*.

**Figure 3:**
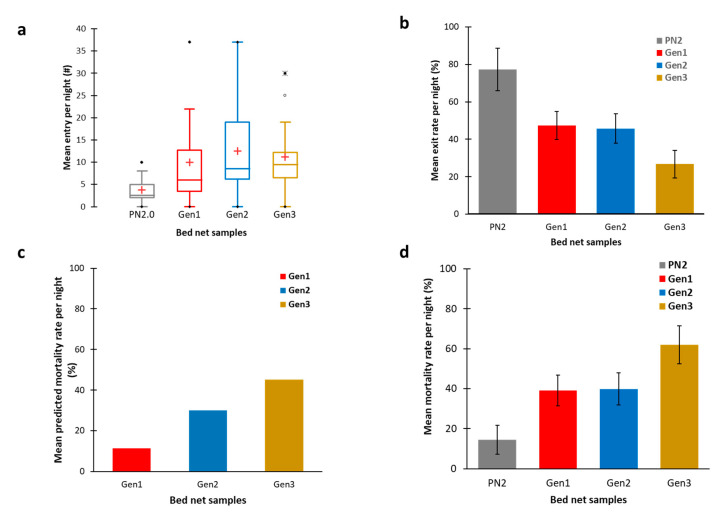
Performance of Gen1, Gen2, and Gen3 insecticide-free T-Nets in comparison to the Permanet 2.0 LLIN (PN2.0) in Tiassale (Cote d’Ivoire) where mosquitoes are resistant to insecticides. The nets were randomly allocated to four huts. Nets and sleepers were rotated each night. Up to 14 observations were made. (**a**) The mean number of mosquitoes entering each hut per night, (**b**) the mean exit rate per hut per night, (**c**) the mean predicted killing rate of the T-Net, and (**d**) the mean mortality rate per hut per night (trapped = dead and ecologically-dead mosquitoes). The bars on top of the histograms represent the 95% confidence intervals.

Mean entry number: A total of 53, 139, 175, and 156 *An. gambiae* s.l. mosquitoes were collected over 14 nights (representing 14 observations = 14 replicates), respectively, for PN2.0, Gen1, Gen2, and Gen3 nets (Table 1). The mean number of mosquitoes entering each hut per night was 3.8 (σ= 2.96) for PN 2.0 (the lowest entry rate in the field trial), followed by 9.9 (σ = 10.1) for Gen1, 12.5 (σ = 10.5) for Gen2, and 11.1 (σ = 8.3) for Gen3. The differences were not statistically significant between Gen1, Gen2, and Gen3, but a significant difference was observed between PN 2.0 and each of the T-Nets (*p* < 0.05) (Figure 3a).

Mean exit rate: As expected, the PN2.0 not only showed the lowest entry rate but also the highest mean exit rate of mosquitoes per night, 77.2% (σ = 29.4) and was statistically significantly different from that of Gen1, Gen2, and Gen3. In Gen1 and Gen2, we recorded a mean mosquito exit rate per night of 47.3% (σ = 27.7) and 45.7% (σ = 30.6), respectively, and in Gen3, the exit rate was 26.7% (σ = 20.7) which was the lowest exit rate. No statistically-significant differences in exit rates were observed between the three T-Nets (Table 1, Figure 3b).

Mean mortality rate: The lowest mortality rate (Figure 3c) occurred with PN2.0, 14.3% (σ= 17.9). It was statistically significantly lower than for all of the T-nets. The mortality rates for Gen1 and Gen2 were 39.1% (σ = 30.5) and 39.8% (σ = 26.0), respectively, with no statistically-significant differences between these two nets (*p* > 0.05). The highest mean mosquito mortality rate per hut per night was for Gen3 at 61.9.5% (σ = 22.1). This difference was higher than that of Gen1, Gen2, and PN.20 (*p* < 0.05).

### 3.4. T-Net Model Validation

Base on the tunnel test, C in Equation (4) was calculated by the condition parameters where the tunnel test cage dimensions were 30 cm in length, 30 cm in width, and 30 cm in height. The cone trap diameter was 3 cm. Thus, C was 0.00783. With these data, we predicted the trap number for Gen1, Gen2, and Gen3, respectively, at 2.87, 1.41, and 5.02 corresponding to trapping rates of 28.9%, 11.3%, and 45.06% (Figure 3c). The relative trap efficiency between Gen1, Gen2, and Gen3 calculated from the model was in agreement but underestimated the actual trap efficacy in the field trials (Figure 3d). This was expected since the model assumptions did not take into consideration the attractants from the sleeper. For the Gen2, there was a much greater trap rate than predicted, suggesting that cone positioning above the sleeper head was likely responsible for a greater number of trapped mosquitoes than the other cones in Gen3. At the same time, the higher efficacy of Gen3 where cones run along the long top axis of the net, suggests that odorants from other parts of the body are important in trap efficacy.

### 3.5. Model for Community-Level Mosquito Control Using the T-Net

**Figure 4:**
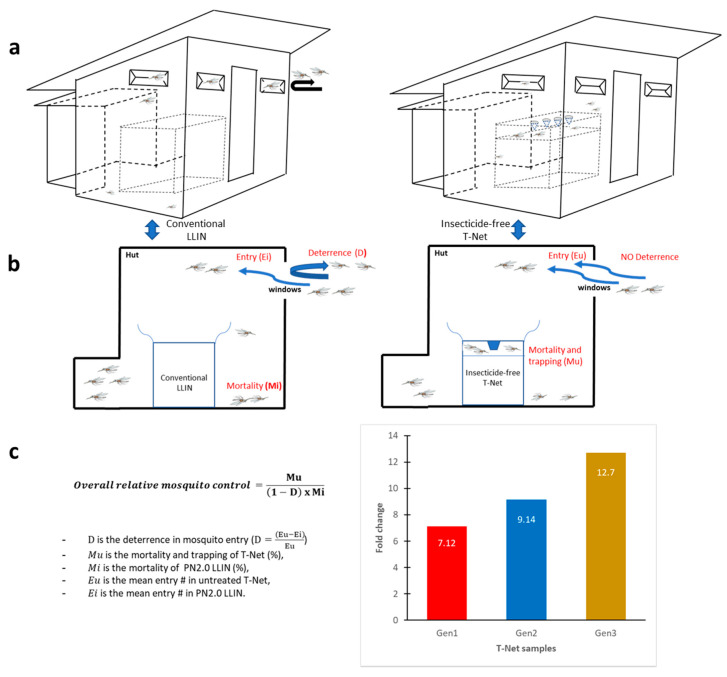
Overall relative mosquito control. (**a**) Schematic illustration of the experimental hut with either a LLIN or a free-insecticide T-net, (**b**) the different parameters used to define the model, and (**c**) overall relative mosquito control. The histograms show the increased mosquito mortality for Gen1, Gen2, and Gen3 T-Nets compared to the PN2.0-LLIN.

Gen1 and Gen2 T-Nets had a 2.7-fold greater kill rate than PN2.0 while Gen3 had a 4.3-fold greater kill rate than the permethrin-treated (PN2.0) positive control. However, there is more to consider in comparing these different bednet technologies, and the T-Net impact at the community level is actually much greater than these data suggest. The deterrence rate (diminution in the rate of entry due to the chemical) was much lower and the repellency rate much higher for PN2.0 compared to the T-Nets (Table 1, Figure 3). If the mosquitoes do not enter the hut because of deterrence and if they enter the hut but are repelled before receiving a lethal insecticide dose from the bednet, these insects are not controlled. The model (Equation (5)) described in Figure 4 takes these factors into consideration to calculate “overall relative mosquito control”:

(5) $$\mathrm{Overall}\;\mathrm{relative}\;\mathrm{mosquito}\;\mathrm{control}\;=\frac{M_{u}}{\left(1-D\right)\;\times \;M_{i}}$$

where$\;M_{u}$ is the mortality caused by the untreated T-Net (%), $M_{i}$ is the mortality caused by the insecticide treated net PN2.0 LLIN (%), D is the deterrence rate$\;\left(D=\frac{\left(E_{u}-E_{i}\right)}{E_{u}}\times 100\%\right),$ $E_{u}$ is the mean entry number for the untreated T-Net, and$\;E_{i}$ is the mean entry number for the insecticide-treated PN2.0 LLIN.

Using this model (5), Gen3 was 12.6-fold more efficacious than PN2.0, and Gen2 and Gen1 were 9.1-fold and 7.1-fold, more efficacious, respectively (Figure 4).

## 4. Discussion

Malaria is a devastating disease, and vector control is a crucial factor in saving lives. However, the situation has become critical because mosquitoes have become resistant to core intervention tools such as LLINs and IRS. Tackling insecticide resistance by developing new approaches for vector control is imperative to controlling malaria, and we need to think “out of the box”.

The current model-driven insecticide-free trapping bednet is a paradigm shift in malaria vector control and could be a simple solution to the problem. Its functionality is based on the attraction and trapping of mosquitoes regardless of their insecticide-resistance status. This proof-of-concept study describes, for the first time, the efficacy of a trapping bednet as a malaria vector control strategy, that addresses the problem of insecticide resistance without the need to develop new chemistry. The advantage of the model developed is that we can predict the mosquito trapping rate before going into the field for testing; this facilitates additional innovation in the future.

As commonly found in experimental hut trials in which non-insecticide and insecticide-treated products are compared [22,23], we found higher mosquito entry rates in huts with T-Nets (free of insecticides) than that observed with the PN2.0-LLIN. Similarly, the exit rate was higher for the PN2.0-LLIN than that of the T-Nets. This is likely due to the excito-repellency property of the insecticide in the nets. The excito-repellency effect can be perceived as beneficial to the extent that it keeps some mosquitoes away from the sleeper, and thus provides personal protection to the net user. However, at the community level, the benefit of the excito-repellency effect can be nullified or even contribute to behavioral resistance and increase outdoor malaria transmission as mosquitoes are repelled outdoors and are not killed [24,25]. Direct observation of our field results showed a 2.7 to 4.3-fold increase mortality for the T-Nets compared to the PN 2.0-LLIN. However, due to mosquito deterrence and repellency for the insecticide-treated bednet, community protection was estimated by our new model to be 13-fold greater for the Gen3 T-Net compared to PN2.0. Thus, mass mosquito trapping from the long-term use of T-Nets could lead to a decline in the vector population where transmission is no longer stable. This population decrease is even more likely in the context of insecticide resistance where mosquito mortality is lower than expected by LLINs.

The T-Net exploits the natural behavior of mosquitoes, which principally interact with the roof of the mosquito net [13,14]. Therefore, the positioning of the traps on the top of the net promotes entrapment of mosquitoes. This is especially true for insecticide-free T-Nets which do not cause repellent effects. Untreated T-Nets can therefore be an asset for vector control as they trap and kill mosquitoes irrespective of their insecticide-resistance status. Nevertheless, the addition of a trap compartment to an existing LLIN can also enhance the killing performance of the net (data now shown). An insecticide-free bednet for malaria vector control is far from being adopted because of regulatory requirements that focus on the use of insecticides in bednets and the possible requirement for epidemiological data to prove the public health value. Although the idea of an insecticide-free bednet for malaria vector control was recently mentioned [26], an interim solution could be a hybrid version of the T-Net consisting of an insecticide-free trap compartment mounted on currently-used LLINs. In this case, community protection would be provided both by the insecticidal effect and the mass trapping of mosquitoes.

Studies have shown that the escalation in resistance to insecticides closely matches the introduction of insecticide-treated nets. To combat resistance to insecticides, the trend today is to use LLINs combined with the synergist piperonyl-butoxide (PBO) [27]. The PBO inhibits enzymatic activity of mosquito insecticide detoxification enzymes, and therefore enhances the insecticidal effect of the treated bednet. Unfortunately, decreases in performance of these mosquito nets are starting to be observed in some regions of Africa where mosquitoes are resistant [22,27]. Because the T-Net is insecticide-free, it does not exert any insecticide-resistance selection pressure on mosquitoes and is not impacted by insecticide resistance. The exclusive and prolonged use of the T-Net in areas that are endemic for insecticide resistance should eventually lead to a decrease in the level of resistance and thus facilitate the reintroduction of insecticides.

Though we have not analyzed the escape rate for trapped mosquitoes in the field, our lab assays suggest the egression rate will be low or zero. We also have found, in laboratory conditions, that trapping elicits a mosquito escape response that rapidly leads to exhaustion and death from dehydration and starvation. The dead mosquitoes in the trap compartment are not easily sighted by someone sleeping under the net, and trapped dead mosquitoes are easily removed by traditional washing methods in Africa. This finding suggests that removing mosquitoes from the T-Net trap compartment is not an issue. They could also be removed by net shaking when the funnels are pulled out. However, we believe that the view of mosquitoes in the trap compartment can be an asset to the extent that it could encourage the use of bednets.

Sampling of mosquito populations for transmission studies or evaluation of other vector-control tools such as IRS could also be conducted with insecticide-free T-Nets. Various anthropophagous mosquito sampling tools exist but are complex to build [28,29,30]. If compared to these methods, the simple construction mode of the T-Net, which uses inexpensive materials, makes it an easy-to-assemble and a less expensive sampling tool. It would be interesting to consider a comparative study of its effectiveness as a sampling method vis-à-vis other methods.

We have not conducted in depth manufacturing cost studies, yet improvements for mass production at low cost are underway. For example, the cones on the roof of the T-Net can simply be mass produced by heat stamping. We anticipate a satisfactory cost range of USD 2–4 [31] per T-Net, which is the current range for LLINs on the market. Furthermore, because there is no new chemistry to develop, the route to market should be rapid compared to an insecticide-treated bednet where new chemistry must be proven safe. It would also be useful to survey public opinion about the use of an insecticide-free bednet versus an LLIN where efficacies for both in a worst-case scenario are equal.

## 5. Conclusions

Proof of concept has demonstrated that a non-insecticidal bednet that kills mosquitoes by mechanical methods, in this case trapping, can be an efficacious “new way of thinking” vastly different from the accepted standard of using bednets treated with insecticides to prevent mosquito biting and to kill mosquitoes in Africa and other places. In this study, the trapping (T)-Net demonstrated a 4.3 fold greater kill rate in experimental huts and a predicted 12.7 fold greater control rate at the community level for insecticide-resistant *Anopheles* mosquitoes than the insecticide treated bednet used as a control. The non-insecticidal T-Net should be considered as another possible tool for malaria control as a stand-alone system, and when constructed from insecticide treated textiles (an insecticide treated T-Net) could be used to improve the efficacy of insecticide treated bednets in general; in both cases, these applications should help reduce the evolution of mosquito resistance to both old and future new insecticide chemistries in bednets.

## 6. Patents

A patent has been awarded in Africa for the T-Net (Patent No. 17063 in BOPI #05BR/2015). Additional IP for the T-Net has been filed by North Carolina State University and is currently pending at the Patent Cooperation Treaty (PCT) in Geneva.

## Figures and Tables

**Table 1:** Results from field evaluations in Tiassale (Cote d’Ivoire) where mosquitoes are resistant to insecticides of Gen1, Gen2, and Gen3 T-Nets compared to the Permanet 2.0 (PN2.0)-LLIN. Shown are the total mosquitoes caught, mean entry rate, mean exit rate, and mean mortality rate per hut per night for *An. gambiae* s.l mosquitoes after 14 observations.

			Entry #		Exit Rate (%)		Killing Rate (%)	
Bednet	# obs.	Total Entry (#)	Mean *	Std dev.	Mean *	Std dev.	Mean *	Std dev.
PN2.0	14	53	3.8a	3.0	77.2a	29.4	14.4a	18.0
Gen1	14	139	9.9b	10.2	47.3b	27.8	39.1b	30.6
Gen2	14	175	12.5b	10.6	45.7b	30.6	39.9b	26.1
Gen3	14	156	11.1b	8.3	26.8b	20.7	61.9c	22.1

# obs., number of observations; * values of the same column not sharing the same letters are statistically significant (*p* < 0.05); Std dev., standard deviation.

## Data Availability

All data generated or analyzed during this study are included in this published article.

## Supplementary Materials

The following are available online at https://www.mdpi.com/2075-4450/11/11/732/s1, Figure S1: Knitted cone fabric and conventional polyester 100-dernier bednet fabric were subjected to an increasing hydrostatic pressure to measure bursting strength. The pressure was applied to a circular region of the fabric sample via an elastic diaphragm. The bursting strength corresponded to the maximum pressure supported by the fabric before failure. (a, left) is the burst strength, (b, middle) is the displacement, and (c, right) is the burst time. The burst strength, displacement, and burst time were higher for the cone fabric than for the bednet textile. Figure S2: Results from the abrasion test on the knitted cone fabric compared to conventional polyester 100-dernier bednet fabric. The assay end point was the duration and number of circles until the first appearance of a hole. The run speed used was 1000 circles (cycles) per 20 min. The abrasion duration of the cone fabric was 8.67 h, corresponding to more than 25,000 circles, whereas for the bednet sample, the duration was 2.67 h and less than 10,000 circles. Thus, the cone fabric presented a better abrasion resistance than the bednet fabric. Figure S3. Ballot box draws were used to assign bednets and sleepers to each hut. Two different Latin-square table designs were used. The first (top) was for bednet rotations, and the second (bottom) was for sleepers. Bednets and sleepers were then rotated according to the above order generated by a Latin-square table generator. Every 4 days, new ballot box draws were conducted to redistribute bednets and sleepers to huts.

## List of Abbreviations

T-Net, trapping bednet; min, minute; WHO, World Health Organization; LLIN, long-lasting insecticidal net; PN2.0, Permanet 2.0; Gen, generation; NCSU, North Carolina State University; Std dev., standard deviation.
