# Supplementary material for: Development of an Insecticide-Free Trapping Bednet to Control Mosquitoes and Manage Resistance in Malaria Vector Control: A New Way of Thinking
Source: Insects. 2020 Oct 26;11(11):732. doi: 10.3390/insects11110732 (PMC7692756; doi:10.3390/insects11110732)
Supplement: Supplementary file 1 [file insects-11-00732-s001.pdf]

Supplementary material

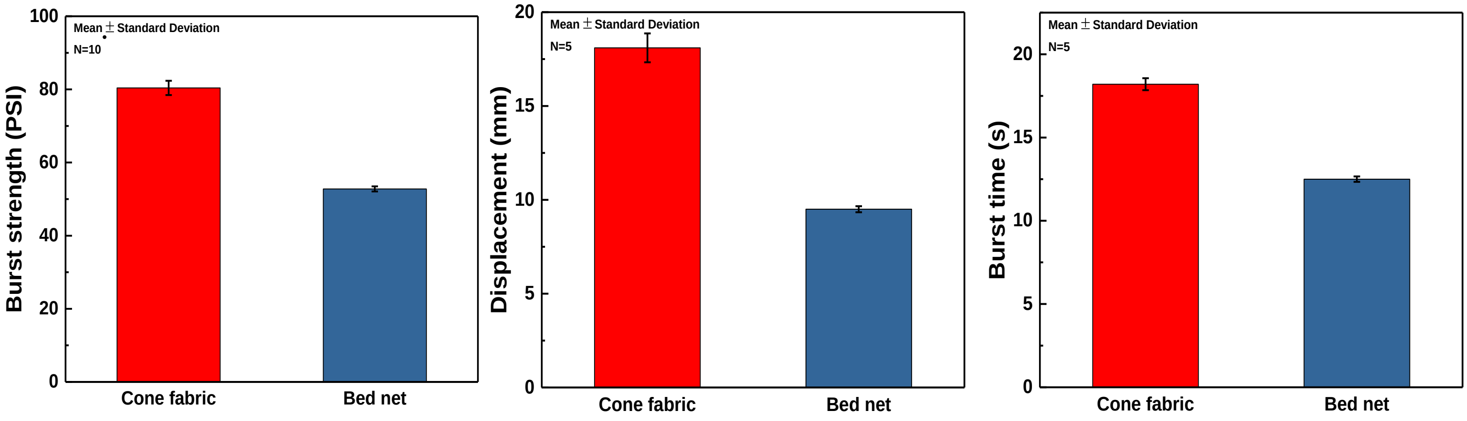

Figure S1. Knitted cone fabric and conventional polyester 100-dernier bed net fabric were subjected to an increasing hydrostatic pressure to measure bursting strength. The pressure was applied to a circular region of the fabric sample via an elastic diaphragm. The bursting strength corresponds to the maximum pressure supported by the fabric before failure. (a, left) is the burst strength, (b, middle) is the displacement and (c, right) is the burst time. The burst strength, displacement and burst time were higher for the cone fabric than for the bednet textile.

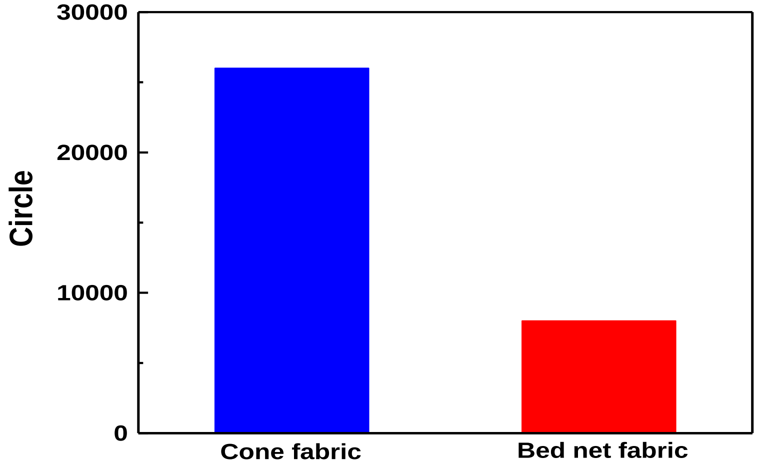

Figure S2. Results from the abrasion test on the knitted cone fabric compared to conventional polyester 100-dernier bed net fabric. The assay end point was the duration and number of circles until the first appearance of a hole. The run speed used was 1000 circles (cycles) per 20 min. The abrasion duration of the cone fabric was 8.67 h, corresponding to more than 25000 circles, whereas for the bed net sample, the duration was 2.67 h and less than 10000 circles. Thus, the cone fabric presented a better abrasion resistance than the bed net fabric.

Bednets	**Hut 1**	**Hut 2**	**Hut 3**	**Hut 4**
**Day1**	Net B	Net A	Net C	Net D
**Day2**	Net D	Net C	Net B	Net A
**Day3**	Net C	Net D	Net A	Net B
**Day4**	Net A	Net B	Net D	Net C
Sleepers				
**Day1**	Slp A	Slp B	Slp D	Slp C
**Day2**	Slp B	Slp C	Slp A	Slp D
**Day3**	Slp C	Slp D	Slp B	Slp A
**Day4**	Slp D	Slp A	Slp C	Slp B

Figure S3. Ballot box draws were used to assign bed nets and sleepers to each hut. Two different Latin square table designs were used. The first (top) was for bed net rotations, and the second (bottom) was for sleepers. Bednets and sleepers were then rotated according to the above order generated by a Latin square table generator. Every 4 days, new ballot box draws were conducted to redistribute bed nets and sleepers to huts.
